# Developmental origins of anti-Black bias in White children in the United States: Exposure to and beliefs about racial inequality

**DOI:** 10.1073/pnas.2209129119

**Published:** 2022-11-15

**Authors:** Michael T. Rizzo, Tobias C. Britton, Marjorie Rhodes

**Affiliations:** ^a^New York University, New York, NY 10003

**Keywords:** racial bias, racial inequality, development, essentialism

## Abstract

Anti-Black racism remains a pervasive crisis in the United States. Racist social systems reinforce racial inequalities and perpetuate prejudicial beliefs. These beliefs emerge in childhood, are difficult to change once entrenched in adolescence and adulthood, and lead people to support policies that further reinforce racist systems. Therefore, it is important to identify what leads children to form prejudicial beliefs and biases and what steps can be taken to preempt their development. This study examined how children’s exposure to and beliefs about racial inequalities predicted anti-Black biases in a sample of 646 White children (4 to 8 years) living across the United States. We found that for children with more exposure to racial inequality in their daily lives, those who believed that racial inequalities were caused by intrinsic differences between people were more likely to hold racial biases, whereas those who recognized the extrinsic factors underlying racial inequalities held more egalitarian attitudes. Grounded in constructivist theories in developmental science, these results are consistent with the possibility that racial biases emerge in part from the explanatory beliefs that children construct to understand the racial inequalities they see in the world around them.

## Significance

Anti-Black racism remains a pervasive crisis in the United States. Understanding the psychological processes and developmental mechanisms underlying the formation of racist thoughts and actions is one important step toward redressing this injustice. Here, White children’s (4 to 8 years) exposure to and beliefs about racial inequalities predicted the development of anti-Black bias. For children with more exposure to racial inequality, those who believed that racial inequalities are caused by intrinsic differences between people were more likely to hold racial biases, whereas those who recognized the extrinsic factors underlying racial inequalities held more egalitarian attitudes. These findings are particularly important in light of legislation in several states banning discussions of the societal factors underlying racial inequalities from children’s classrooms.

In the United States today, the perpetuation of racial bias, prejudice, and discrimination maintain and reflect racist social systems that systematically advantage White people while systematically disadvantaging Black, Indigenous, and other people of color (1–3). One critical step to redressing this injustice is understanding the psychological processes that lead to the formation of racist thoughts and actions (4, 5). However, little is known about the developmental mechanisms that underlie the emergence of these thoughts and actions. Given the scale and scope of racial bias in the United States, there are undoubtedly multiple social and psychological processes involved; for example, experiences with intergroup contact explains ∼4% of variation in bias (5, 6). In the present study, we examined an additional mechanism that we hypothesize underlies the development of anti-Black bias in White children living across the United States: children’s exposure to and explanatory beliefs about racial inequalities. We expected that children who have had more exposure to racial inequalities in their daily lives (i.e., older children living in neighborhoods with larger disparities between Black and White people) would hold racial biases reflective of their beliefs about those inequalities. Specifically, we expected that children who believe that racial inequalities are caused by intrinsic differences between people would have more racial biases because they are coming to view race and status as inherently linked, whereas children who believe that inequalities are caused by extrinsic factors would have fewer racial biases because of the recognition that it would be unfair and inaccurate to judge people based on these extrinsic circumstances. To shed light on this mechanism, we assessed children’s endorsement of intrinsic and extrinsic explanations for racial inequalities, children’s exposure to racial inequality in their residential ZIP code (i.e., Black/White disparities in income and education with children’s age as a measure of the duration of exposure), and children’s racial biases using two standard assessments of bias in childhood (i.e., children’s choice to play with and attitudes about peers from different racial backgrounds (7–14)). We then examined how children’s exposure to and beliefs about racial inequalities interact to predict variation in anti-Black bias during childhood.

Explicit anti-Black biases emerge early and continue to develop throughout childhood (12). By 4 to 5 years of age, children—and White children in particular—hold more negative attitudes toward Black than White peers, attribute more negative intentions to Black than White peers, and are less likely to form friendships with Black than White peers (7–14). These biases are consequential. The majority of Black children and adolescents report experiencing racial prejudice and discrimination and suffer from increased stress, decreased social belonging, and restricted educational and extracurricular opportunities because of these experiences (15–17). Early emerging biases also serve as the developmental roots for the racial bias, prejudice, and discrimination that become deeply entrenched in adolescence and adulthood and lead people to support racist social policies (18–20). Importantly, there is also substantial individual variation in the early development of racial bias; some children develop more racial biases than do others (12, 13). Early childhood is therefore an ideal time for disrupting the formation of problematic beliefs; by understanding the predictors of this individual variation (i.e., why some children develop more biases than others) at a time when children’s beliefs are emerging and particularly sensitive to new experiences, we can better inform future efforts to understand and disrupt the formation of racial biases before they become deeply entrenched.

In the present study, we used an online platform for conducting remote, unmoderated psychological research with children (21) to examine the environmental and psychological factors that predict the development of anti-Black bias in a sample of 646 White children 4- to 8-years-old (300 female, 346 male) living across the United States. We focused on White children in this age range because they are particularly likely to develop anti-Black biases and hold disproportionate power and influence over social contexts by virtue of their racial privilege (3–5, 12, 13). Children participated from 464 unique ZIP codes representing 47 different states; this degree of cultural, geographic, and socioeconomic diversity allowed for an analysis of the variance in children’s environment that is not possible with traditional, in-person approaches to developmental science. Using the online platform, children completed the study independently from their home computer without interacting with a researcher, reducing concerns for self-presentation. Research sessions were recorded via the computer’s webcam for subsequent coding.

## Results and Discussion

**Fig. 1.**
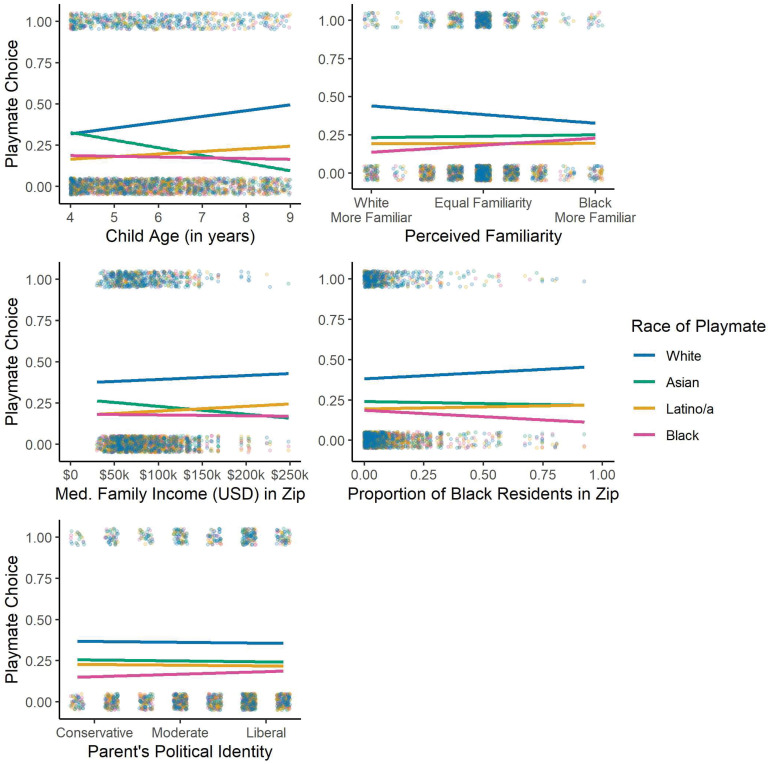
Proportion of children choosing playmates of each racial group (chance = 0.25; blue: White, green: Asian, yellow: Latino/a, pink: Black) across child (age, perceived familiarity), neighborhood (median family income, proportion of Black residents), and parent (political identity) factors. Lines represent linear regression lines for each racial group. Dots represent participants’ responses.

**Fig. 2.**
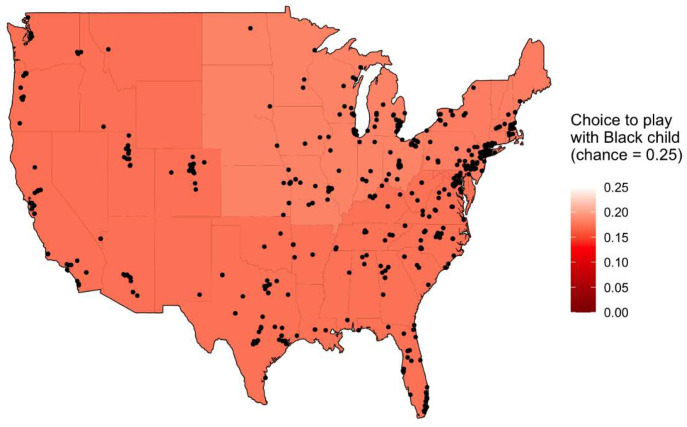
Map of regional variation in children’s choice to play with the Black child. Darker red reflects more anti-Black bias (i.e., lower likelihood of choosing the Black child), the white of the page reflects chance levels of choosing to play with the Black child. Black dots represent participants’ approximate geographic location.

Our primary focus for this study was the emergence of anti-Black bias in White children’s playmate preferences (i.e., who they want to play with) given the immediate consequences of racial discrimination and exclusion for Black children (15–17), the connection between an early lack of interracial contact and the long-term formation of racial bias (6, 22), and the increased likelihood for White children to develop anti-Black biases in the United States (9, 12, 13). Building on past research using this measure of bias (7–14), we presented participants with photographs of 4 gender-matched children (1 Asian, 1 Black, 1 Latino/a, 1 White) using fully narrated and animated prompts and asked them who they wanted to play with the most. Consistent with past research, children were least likely to choose to play with the Black child and did so significantly less often than expected by chance (chance = 0.25; mean = 0.18; χ^2^ = 16.98, *df* = 1, *P* < 0.001). Children also chose to play with the Latino/a child less often than expected by chance (mean = 0.19, χ^2^ = 10.21, *df* = 1, *P* = 0.001), the Asian child at chance levels (mean = 0.24, χ^2^ = 0.26, *df* = 1, *P* = 0.61), and the White child more often than expected by chance (mean = 0.39, χ^2^ = 61.22, *df* = 1, *P* < 0.001). Importantly, the geographic diversity of our sample allowed us to expand on past research by examining several environmental and psychological factors that may relate to children’s racial biases. As shown in Figs. 1 and 2, multinomial regressions revealed that children’s choice to play with the Black child was not related to their age (all *P* > 0.052), their perceived familiarity of Black and White peers (all *p*s > 0.18), their parents’ political identity (all *P* > 0.61), the median family income or proportion of Black residents in their residential ZIP code (all *P* > 0.13 and 0.45, respectively), or the region of the United States in which they lived (all *P* > 0.60).

These results document the ubiquity of anti-Black racial bias in White children living across the United States. However, a critical question remains: how do racial biases develop? From a constructivist perspective on developmental science, children learn by observing patterns in the world and actively constructing explanations about the causal structure underlying those patterns (23). These observations and interpretations inform children’s mental model of the world and in turn guide how they think and act within it. Accordingly, to understand how children develop anti-Black bias, we need to identify (1) the race-related patterns that children observe in the world around them, (2) the beliefs they develop to explain those patterns, and (3) how those beliefs relate to children’s developing anti-Black bias.

Children growing up in the United States today are embedded within a society that is structured by erroneous notions of White supremacy that reinforce racist social systems that systematically advantage White people and disadvantage people of color—and Black people in particular (1–5). In the 2020 US Census, the median family income was 63% higher for White than Black families ($74,912 and $45,870, respectively) and White adults were 46% more likely to have a Bachelor’s degree than Black adults (41% and 28%, respectively) (24). Our sample reflected these inequalities. The average median family income across our participants’ residential ZIP codes was 44% higher for White than for Black families ($86,731 and $60,100, respectively) and White adults in these neighborhoods were over twice as likely to have a Bachelor’s degree than Black adults (47% and 22%, respectively). Children become aware of these inequalities by 4 to 5 years of age (25, 26). Indeed, when we presented children in this sample with photos of Black and White children and high- and low-wealth housing, the majority (64.2%; different from chance: χ^2^ = 47.35, *df* = 1, *P* < 0.001) expected the White child to live in the high-wealth housing and the Black child to live in the low-wealth housing, suggesting that children are aware of the racial inequalities they observe in the world around them.

Awareness of racial inequalities is not necessarily problematic, however, and may in fact be a prerequisite for understanding and redressing systemic barriers to racial equity (3–5, 18, 19, 27, 28). What likely matters for the formation of anti-Black bias are the beliefs that children develop to explain why racial inequalities exist. One problematic explanatory belief that may develop is that racial inequalities arise because of intrinsic or essential differences between racial groups (13, 29, 30). That is, children may believe that racial groups reflect fundamentally distinct kinds of people, with White people having intrinsically higher status (e.g., harder working, more intelligent) and Black people having intrinsically lower status. Essentialist beliefs like these contribute to bias and prejudice and may lead children to avoid playing with Black peers if they perceive them to be intrinsically lower status (13, 31, 32). Alternatively, children may develop the explanatory belief that racial inequalities arise because of extrinsic differences in how racial groups are treated within society. Children who develop this belief may in turn be more likely to play with their Black peers because they recognize that it would be unfair and inaccurate to negatively evaluate them based on these extrinsic factors. Indeed, adolescents who have a better understanding of the external, societal barriers imposed on marginalized groups are more likely to engage in social action to redress social inequalities (28). Thus, exploring how children’s explanatory beliefs shape their racial attitudes as they are first developing may yield critical insights into how to disrupt the formation of prejudicial beliefs before they become deeply entrenched.

To address this question, we presented children with a short vignette about a Black child who lived in a low-wealth house and a White child who lived in a high-wealth house and asked them whether they thought they lived in these houses due to intrinsic (“because of who they are on the inside”) or extrinsic (“because of things that happen in the world”) factors. Children indicated how true or not true they thought each explanation was on a 5-point Likert-type scale (1 = “really not true” to 6 = “really true”). Overall, children endorsed the extrinsic (mean = 3.56, SD = 1.49) over the intrinsic (mean = 3.39, SD = 1.56) explanation (*t* = 2.00, *df* = 604, *P* = 0.046), although there was significant variation across children, with 34% (*n* = 205) endorsing the extrinsic over the intrinsic, 26% (*n* = 160) endorsing the intrinsic over the extrinsic, and 40% (*n* = 240) endorsing both explanations to the same extent.

**Fig. 3.**
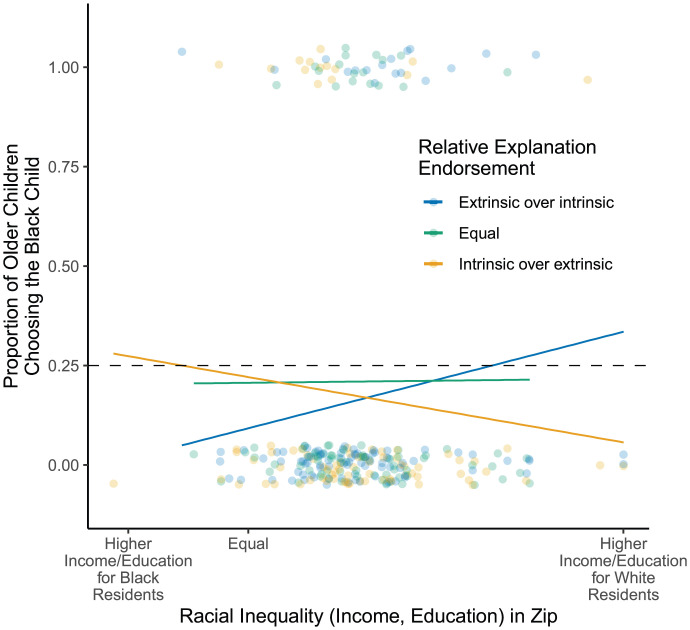
Proportion of older children (i.e., children above the median age; 5.62 to 8.99 years) choosing to play with the Black child (y axis) by the degree of racial inequality in children’s residential ZIP code (standardized composite of Black/White racial inequality in family income and college graduation rates; x axis) and children’s relative endorsement of the intrinsic and extrinsic explanations for racial inequalities (legend; analyses were conducted on the continuous scale for relative endorsement, groups were defined for data visualization purposes). Lines represent the linear regression line for each explanation endorsement group and dots represent participants’ responses (blue: endorsed extrinsic over intrinsic, green: endorsed extrinsic and intrinsic explanations equally, yellow: endorsed intrinsic over extrinsic). The hashed black line represents the level at which children would be expected to choose to play with the Black child by chance. Follow-up analyses revealed no significant effects for younger children (i.e., children below the median age; 4.00 to 5.61 years; all *P* > 0.12).

Critically, children’s endorsements of these explanations were related to their choice to play with a Black child, and particularly for those who have had more exposure to racial inequalities in their daily lives (i.e., older children living in residential ZIP codes with larger Black/White racial disparities), where children’s explanations for inequality are more relevant for understanding their daily experiences*. To assess children’s exposure to racial inequality, we used a standardized composite of the Black/White racial inequalities in median family income and college graduation rates within participants’ residential ZIP codes and used children’s age as a measure of their time spent in these contexts (1). As shown in Fig. 3, for children with more exposure to Black/White racial inequality (i.e., older children living in residential ZIP codes with larger Black/White racial disparities), those who endorsed the intrinsic over the extrinsic explanation were less likely to choose to play with the Black child, whereas those who endorsed the extrinsic over the intrinsic explanation were more likely to choose to play with the Black child (age by explanation by exposure interactions: Black-Asian contrast: β = 0.18, SE = 0.07, *P* = 0.008; Black-Latino/a contrast: β = 0.22, SE = 0.07, *P* = 0.002; Black-White contrast: β = 0.14, SE = 0.06, *P* = 0.012). Importantly, these effects held while controlling for various neighborhood factors (e.g., proportion of Black residents, median family income, US region; all Black-Asian contrasts *P* < 0.008; all Black-Latino/a contrasts: *P* < 0.002; all Black-White contrasts *P* < 0.014; all neighborhood controls *P* > 0.37). To explore the interaction with age, we ran separate models for younger and older children using a median split (median age = 5.62 years) that revealed that these effects were driven by older children (all *P* < 0.042). No significant effects were found for younger children (all *P* > 0.12).

We then conducted follow-up analyses testing children’s endorsement of the extrinsic and the intrinsic explanations separately. Consistent with the relative endorsement results discussed above, for children with more exposure to Black/White racial inequality, those who endorsed the extrinsic explanation to a greater extent were more likely to choose to play with the Black child (age by explanation by exposure interactions: Black-Asian contrast: β = −0.32, SE = 0.11, *P* = 0.003; Black-Latino/a contrast: β = −0.29, SE = 0.11, *P* = 0.010; Black-White contrast: β = −0.21, SE = 0.09, *P* = 0.023). Conversely, those who endorsed the intrinsic explanation to a greater extent were less likely to choose to play with the Black child relative to the Latino/a child (β = 0.22, SE = 0.09, *P* = 0.017); the contrasts between Black and Asian (*P* = 0.096) and Black and White (*P* = 0.064) followed similar patterns but were not significant. Taken together, these results are consistent with our hypothesis that as White children’s exposure to Black/White racial inequalities in their daily lives increases, the beliefs they develop to explain those inequalities become particularly important for informing their choice to play with their Black peers. Children who recognize the extrinsic, societal factors that underlie racial inequalities are more likely to choose to play with a Black child, whereas those who attribute racial inequalities to intrinsic differences between people are less likely to choose to play with a Black child.

As stated above, our primary focus for this study was the emergence of anti-Black biases in White children’s playmate preferences. However, racial biases manifest in many ways in early childhood, including stereotypes, hostile attributions, and implicit and explicit racial attitudes (7–14). Therefore, we also collected data assessing how positively or negatively children rated Black and White peers more generally.

To do so, we showed children new pictures of Black and White children (age and gender matched) and asked them to rate how nice they thought each child was, one at a time, on a 6-point scale (1 = “really not nice” to 6 = “really nice”). We did not find evidence of bias across the full sample on this measure (Black: mean = 5.09, SD = 1.29; White: mean = 5.03, SD = 1.27; *P* = 0.42). Consistent with past research (12, 13), however, younger children were more likely than older children to perceive White children as nicer than Black children (main effect for age: β = 0.16, SE = 0.047, *P* < 0.001) and children who perceived White children as nicer than Black children were less likely to choose to play with the Black child in the playmate preferences assessment (*r* = 0.09, *P* = 0.037). Furthermore, consistent with their playmate preferences, we did not find differences in children’s niceness ratings by parents’ political identification, US region, or the median family income or proportion of Black residents in their residential ZIP codes (all *P* > 0.09).

**Fig. 4.**
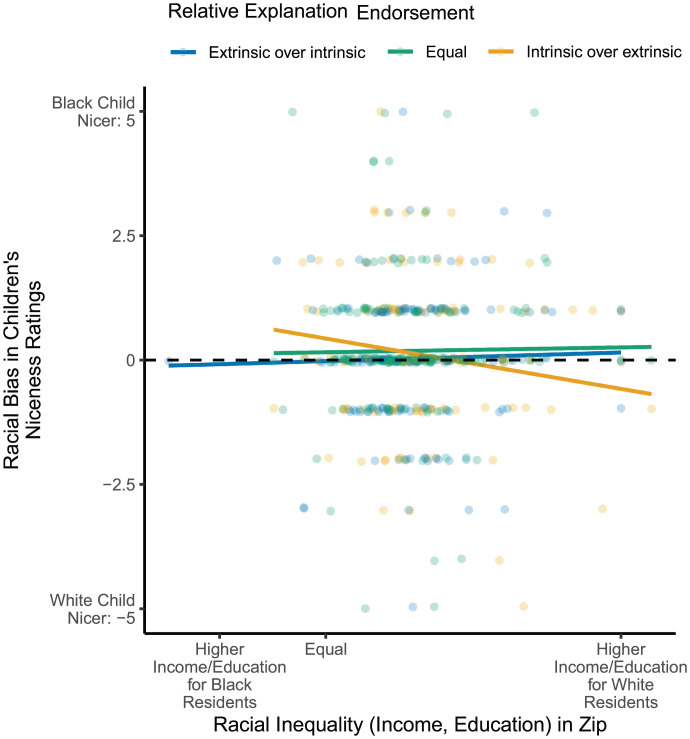
Racial bias in children’s niceness ratings of Black and White children (y axis) by the degree of racial inequality in children’s residential ZIP code (standardized composite of Black/White racial inequality in family income and college graduation rates; x axis) and children’s relative endorsement of the intrinsic and extrinsic explanations for racial inequalities (legend; analyses were conducted on the continuous scale for relative endorsement, groups were defined for data visualization purposes). Lines represent the linear regression line for each explanation endorsement group and dots represent participants’ responses (blue: endorsed extrinsic over intrinsic, green: endorsed extrinsic and intrinsic explanations equally, yellow: endorsed intrinsic over extrinsic). The hashed black line represents equal niceness ratings of Black and White children.

We next explored whether children’s exposure to and explanatory beliefs about racial inequalities predicted variation in children’s bias on this measure in a similar manner as their playmate preferences. Indeed, as shown in Fig. 4, among children with greater exposure to Black/White racial inequalities (i.e., children living in residential ZIP codes with larger Black/White racial disparities, in this case, regardless of age), those who endorsed the extrinsic explanation to a greater extent were less likely to rate White children as nicer than Black children (exposure by explanation interaction: β = 0.12, SE = 0.06, *P* = 0.045). On this measure, we only found effects for children’s endorsement of the extrinsic explanation; we did not find comparable effects of endorsement of the intrinsic measure or children’s relative endorsement. Variation across these different aspects of racial bias should be interpreted cautiously, however, given possible differences in measurement sensitivity (13, 33). Nevertheless, these results provide additional, converging evidence that children’s exposure to and beliefs about racial inequalities are related to the development of broader anti-Black biases (and suggest that these effects are not specific to children’s playmate preferences or idiosyncratic features of the photographs selected for the assessments).

Overall, our findings provide important insights that are consistent with the account of the emergence of racial bias in early childhood outlined above. Racial biases emerge in part from the explanatory beliefs that children construct to understand their social environment. This account illuminates the dynamic and self-perpetuating nature of racist social systems. Racist systems create racial inequalities and perpetuate myths that those inequalities are caused by intrinsic differences in work ethic, ability, or intelligence (1–5). The present results are consistent with the idea that, as children observe inequalities in the world around them, those who endorse the myths about intrinsic differences in turn develop biases that reinforce racist systems, whereas those who recognize the extrinsic factors underlying racial inequalities develop more egalitarian attitudes. Broadly speaking, these results highlight the importance of teaching children—and White children in particular—about the extrinsic, systemic factors that underlie racial disparities and support calls to address problematic beliefs about racial inequalities early in development (18, 19, 34, 35). Early intervention may be especially important given that beliefs are often more malleable as they are emerging (23). Future research is still needed, however, to identify effective means for intervening on these beliefs during childhood (e.g., parent–child conversations, school-based interventions, media-based interventions). This future work is particularly important in light of legislation in an increasing number of US states banning discussions of the societal factors underlying racial inequalities in children’s schools (36).

Importantly, given the correlational nature of the present study, it is also possible that children with more anti-Black biases are subsequently more likely to attribute racial inequalities to intrinsic differences between racial groups, whereas children with less anti-Black bias are subsequently more likely to reason about extrinsic factors. Although future work is undoubtedly needed to test this possibility directly, we believe this causal direction is less likely for two reasons. First, the present findings are consistent with longitudinal work revealing that 4 year old's explanatory beliefs about racial inequalities predict the development of racial bias over time (13). The present study substantially builds on this work by revealing the interaction between children’s exposure to and beliefs about racial inequalities in a large sample of children from across the United States. Second, our findings are consistent with prior experimental work with novel (fictional) social categories suggesting that inducing children to hold intrinsic or extrinsic explanatory beliefs about group differences has immediate consequences for the expression of social biases (37–39). Experimental studies with novel groups can confirm the causal connection between explanatory beliefs and social bias, something that is not possible with the present correlational data, but cannot speak to whether effects of explanatory beliefs are powerful enough to shape how children respond to the much more complicated categories they encounter in their daily lives. The present findings indicate that the effects of explanatory beliefs about social inequalities are powerful enough to predict variation in racial bias as it emerges in daily life, which is striking given all of the complicated and variable experiences that children have related to race (in comparison to the highly simplified and constrained experiences with novel categories in experimental studies).

Children’s racial biases did not relate to their parents’ political identification in the present study. Past research has found that adults’ political identities often relate to their racial attitudes (40) and that parents’ and older children’s racial attitudes often relate to one another (41). It is possible that the link between parents’ and children’s racial attitudes develops later in childhood or adolescence or that measuring parents’ racial attitudes directly would have yielded different results. Either way, our results indicate the importance of future work examining the role of parents’ racial socialization practices with White children (34, 42) and how these practices are moderated by additional environmental factors.

These findings highlight the importance of using a large and geographically diverse sample, examining the interplay between environmental and psychological factors, and using multiple assessments of bias to identify the developmental mechanisms underlying the emergence of racial bias. Indeed, an important limitation of the existing literature is the use of smaller sampling populations that do not allow for an analysis of environmental variation in the development of children (21). However, there are still limitations to our approach. For example, although the playmate preferences assessment was chosen based on its extensive use within the developmental literature (7–14), a limitation of this assessment is that it only measures children’s choices in a single trial, making it possible that children’s individual choices were influenced by idiosyncratic differences in the photographs used. The correlation between and consistent pattern of results across the playmate preferences and niceness rating assessments (which used different photographs) makes this less likely. Nevertheless, future work should address this limitation by assessing children’s playmate preferences across multiple trials and including additional measures of bias.

Finally, it will be critical for future work to use this broader sampling approach to examine the development of biases against other racial groups (e.g., anti-Asian racism, anti-Latino/a racism, and racism directed at native and indigenous populations), biases against other social categories (e.g., gender, sexuality), and intersectional biases across social categories (e.g., Black female invisibility) (43, 44). Our research suggests that children’s exposure to and beliefs about Black/White racial inequalities specifically predict anti-Black racial biases, but that these beliefs and experiences do not predict children’s biases toward other groups per se. As such, future research should consider the specific beliefs and experiences underlying biases directed at different populations. Longitudinal investigations would be particularly valuable for identifying unique predictors of bias, as well as potential mediating variables between children’s experiences, beliefs, and biases. Importantly, although our findings highlight the meaningful role that psychological and developmental science can play in redressing societal injustices, we also note that disrupting biased systems through economic, political, legal, and other societal means is undoubtedly necessary to achieve broader social justice.

## Materials and Methods

### Participants and Procedure.

Data for the present study were drawn from the first wave of an ongoing longitudinal study on the development of multiple components of racial bias in children from diverse racial backgrounds living across the United States. All monoracial White participants who completed the full version of the first wave of the ongoing longitudinal study between November 11, 2020 and January 30, 2022 were included in these analyses. The preregistration and full list of measures for the ongoing longitudinal study can be found at: https://osf.io/8cr2u/. The analyses for the present paper were not preregistered.

Children (*n* = 646; female: *n* = 300, male: *n* = 346) ages 4 to 8 years old (mean*_age_* = 5.85, SD*_age_* = 1.38, min*_age_* = 4.00, max*_age_* = 8.99) participated from 47 states (Midwest: *n* = 126, Northeast: *n* = 201, Pacific: *n* = 1, South: *n* = 178, West: *n* = 120; did not report: *n* = 20) representing 464 unique ZIP codes across the United States (ZIP code demographics: mean*_population_* = 30,921, SD*_population_* = 18,338, min*_population_* = 447, max*_population_* = 114,341; mean*_median_family_income_* = $83,255, SD*_median_family_income_* = $33,910, min*_median_family_income_* = $30,093, max*_median_family_income_* = $248,088). Parents indicated their political identification on a separate survey asking them to report their overall political identification on a scale from: 1 = “very conservative” to 7 = “very liberal.” A total of 103 parents identified as conservative, 74 identified as moderate, and 228 identified as liberal (241 parents did not complete the survey or did not indicate their political identification). All of the participants spoke English.

Children completed the study using their home computer via a virtual laboratory developed for unmoderated remote developmental research (21). Participants were recruited to the virtual laboratory via social media advertising, community outreach events, parenting podcasts and blogs, and volunteer sign-ups at in-person data collection sites. All children between 4 and 8 years old that were registered on the virtual laboratory during the period of data collection were invited to participate.

The full study protocol was approved by the institutional review board at New York University (study ID: IRB-FY2020-4503; title: Developmental Mechanisms Underlying the Emergence of Racial Bias). Parents indicated consent by providing a signature onscreen or by repeating a consent script out loud, and children provided assent by pressing a child-friendly “Yes” button or verbally indicating their assent to participate. The full experimental session was recorded via the participant’s webcam and uploaded to the virtual laboratory at the end of the research session for subsequent coding (see Video Coding for Interference section below); survey data (e.g., button responses) were collected via Qualtrics using fully animated and narrated question prompts. Following the consent and assent procedures, parents were instructed to not intervene on children’s responses and were asked to save all questions/comments for their child until after the study. Children indicated their responses to the study assessments by clicking on the response buttons (smiling/frowning and thumbs up/down Likert-type scales). All of the procedures were developed and piloted to ensure that children as young as 4 years old could independently complete the study. In total, the sessions took ∼15 min to complete. At the end of the session, families were compensated with a $10 Amazon gift card for their participation.

The photographs of children’s faces used throughout the study were selected from a database of 476 photographs of Asian, Black, Latino/a, and White children between 4 and 8 years old that were normed on MTurk for perceived age, affect, attractiveness, and wealth status. A total of 475 MTurkers rated a random selection of 50 photographs, such that each photograph was rated by an average of 49 MTurkers (minimum = 42, maximum = 57). The photographs used in each assessment were chosen based on a match in the MTurkers’ perceived age, affect, attractiveness, and wealth status and the complete agreement of 3 research assistants regarding the perceived race of each child (i.e., all three raters agreed on the perceived race of the child). Different photographs were used for each bias assessment.

### Measures.

Videos illustrating all measures are available at https://osf.io/q8b7g/.

#### Playmate Preferences.

Participants were presented with an array of 4 photographs of gender-matched children from different racial backgrounds (1 Asian, 1 Black, 1 Latino/a, and 1 White) and were asked to choose who they would like to play with the most. Responses were scored as “Asian,” “Black,” “Latino/a,” and “White.”

#### Niceness Ratings.

Participants were presented with photographs of one Black and one White child (sequentially, randomized order) and were asked: “How nice do you think this kid is?” Participants indicated their responses on a 6-point Likert-type scale: 1 = “really not nice” to 6 = “really nice.” A difference score was created by subtracting children’s rating for the White child from their rating for the Black child (i.e., nice_Black − nice_White). The full range for the composite score was thus −5 = maximal “White-nicer” bias to 5 = maximal “Black nicer” bias.

#### Perceptions of Familiarity.

Using the same images in the niceness ratings task, participants were asked, “Do you think you might see this kid in your neighborhood?” Participants indicated their response on a 5-point Likert-type scale: 1 = “definitely not” to 5 = “definitely yes.” A difference score was created by subtracting children’s perceived familiarity for the Black child from their perceived familiarity for the White child (i.e., familiar_White − familiar_Black). The full range for the composite score was thus −4 = maximally more familiar with Black children to 4 = maximally more familiar with White children.

#### Awareness of Racial Inequalities Task.

This task was adapted from previous research (13, 25, 26). Participants were shown photographs of a high-wealth house, a low-wealth house, a Black child, and a White child and were told that each of the children lived in one of the houses. Participants were then asked which house they expected each child to live in. Responses were scored as “1” if participants indicated that the White child lived in the high-wealth house and the Black child lived in the low-wealth house and a “0” if participants indicated that the Black child lived in the high-wealth house and the White child lived in the low-wealth house.

#### Explanations for Racial Inequalities Task.

This task was adapted from previous research (13). Participants were presented with a unique set of photographs of a high-wealth house, a low-wealth house, a Black child, and a White child, with the White child overlaid onto the high-wealth house and the Black child overlaid onto the low-wealth house. Participants were then told that the White child lived in the high-wealth house and that the Black child lived in the low-wealth house (wiggle animations played while the narrator said, “This kid lives in this house and this kid lives in this house”) and were asked to think about why they lived in those houses. Children were then told about two explanations ostensibly provided by other children: extrinsic explanation (“One kid said that this one lives in this house and this one lives in this house because of things that happen in the world. They said that there are things people don’t have any control over that make it harder for some kids and easier for others, and it’s these things that happen that make it so that each of these kids lives in these houses.”) and intrinsic explanation (“Another kid said that this one lives in this house and this one lives in this house because of who they are on the inside. They said that there are things about who people are that make it so that there are different types of people in the world, and who these kids are on the inside makes it so that each of these kids live in these houses.”). Children were then asked how much they agreed that each explanation was true on a 5-point Likert-type scale: 1 = “definitely not” to 6 = “definitely yes.” There was a small correlation between the explanations (*r* = 0.14, *P* < 0.001). A difference score was then created for analyses by subtracting children’s extrinsic endorsement from their intrinsic endorsement (i.e., intrinsic_endorsement − extrinsic_endorsement). The full range of the composite was thus −4 = maximal extrinsic endorsement to 4 = maximal intrinsic endorsement.

To avoid perpetuating race-wealth stereotypes within the study, children were debriefed with a similar vignette about a Black child living in a high-wealth house and a White child living in a low-wealth house.

#### Neighborhood Demographic Data.

Demographic information for children’s residential ZIP codes was pulled from the 2019 US Census American Community Survey (ACS), which was the year the longitudinal project began. ACS data were pulled at the ZIP Code Tabulation Area level and then transformed to ZIP code. Analyses testing differences by US region used the regional classifications provided by the US Census Bureau (Midwest, Northeast, Pacific, South, West).

#### Racial Inequality Index.

A composite score was created to capture the degree of racial inequality in participants’ residential ZIP codes across two domains with well-documented disparities: economics and education (1–5). Specifically, we standardized the difference scores between Black and White residents’ median family income and college graduation rates, and then averaged across those standardized difference scores to create an overall racial inequality index score.

### Video Coding for Interference.

All of the research sessions were video and audio recorded using the camera and microphone on the participants’ computers. In a first wave of coding, research assistants checked the recordings to ensure that the parent and child provided consent and assent to participate, and that the child remained at the computer for the duration of the study. If parents and children did not provide consent or assent to participate, or if the child was not present to respond to the assessments, then their data were not included in the sample. A second wave of coding was conducted on a subset of the data (*n* = 165; 26%) to assess the frequency of external interference on children’s responses (e.g., parents/siblings influencing children’s responses to the assessments). Research assistants were trained to identify instances in which external influences may have affected children’s responses (e.g., parents/siblings commenting on the protocol/stimuli, indicating whom to choose, indicating whom they would choose). Consistent with other research using the virtual laboratory (21, 45), interference rates were very low. Interference was found for only two participants (one parent interference, one sibling interference) and thus was unlikely to alter the study findings (indeed, patterns were identical with those two participants excluded from the dataset). Thus, consistent with established procedures of the virtual laboratory, all of the data were retained for analyses.

Descriptively, the typical recording was of a child independently completing the study with parents/siblings observing from the other side of the room or completing other tasks in the home. For younger participants, parents often sat next to the child during the study and complied with the instructions to not discuss their child’s answers until the end of the study.

## Data Availability

Anonymized (deidentified data, R script, protocols) data have been deposited in OSF (https://osf.io/q8b7g/).

## Footnotes

^*^Analyses including the degree of racial inequality in participants’ residential ZIP codes were conducted on a subset of participants (*n* = 563) whose residential ZIP code had at least 100 Black and 100 White residents; this was done to ensure reasonable estimates for Black and White residents’ median family income and proportion of Black and White residents receiving a college degree. Significant effects were not found when examining the full sample, suggesting that these results only hold in communities with at least 100 Black and 100 White residents.
